# Insights from the fifth International One Health Congress, 2018, Saskatoon, Canada

**DOI:** 10.11604/pamj.2019.32.168.16542

**Published:** 2019-04-09

**Authors:** Anelisa Jaca

**Affiliations:** 1 Cochrane South Africa, South African Medical Research Council, Cape Town, South Africa

**Keywords:** Emerging diseases, vaccination, antimicrobial resistance

## Abstract

The recent outbreaks of diseases including Ebola reemergence in the Democratic Republic of Congo (DRC), Nipah virus outbreak in India, Lassa virus in Nigeria and the continued Influenza pandemic show that we cannot predict outbreaks, however, developing response plans could help alleviate the burden of diseases. Developing response plans involves strategies such as creating a repository of microbial agents as well as the implementation of the plans. In addition, zoonotic experts and policy makers should work together in order to succeed in fighting against any disease outbreaks. We should also not forget about the importance of vaccination because of the benefits it has brought to humankind preventing disease occurrence. Furthermore, the challenges that come with vaccination including vaccine delivery and vaccine uptake need to be overcome to make sure that this public health tool continues to be effective. Overcoming these vaccination challenges would play a significant role in decreasing the overuse of antimicrobials, hence avoiding resistance. The One Health Community (OHC) therefore has the responsibility to advocate for the use of vaccines and show that it has costs benefits; this strategy has the potential to fight against antimicrobial resistance.

## Introduction

The fifth International One Health Congress was held in Saskatoon, Canada and was tailored towards discussing the threats of emerging and reemerging diseases, addressing the challenges the world faces concerning antimicrobial resistance and bridging the gap between zoonotic experts and policy makers in the field of disaster risk reduction. Regarding emerging and reemerging diseases, there was a discussion on four major disease recent outbreaks including Ebola reemergence in the Democratic Republic of Congo (DRC) [1], Nipah virus outbreak in India [2], Lassa virus in Nigeria [3] and the continued Influenza pandemic [4]. With regards to the emerging and reemerging diseases, the question that was posed was: can we predict major disease outbreaks? It was clear from the reemergence of those outbreaks that we are not able to predict major disease outbreaks. Therefore, since we are not able to predict those epidemics, we can prepare in advance to counter the emergence of diseases by developing preparedness or response plans which will assist in managing and preempting the spread of the specific disease. Furthermore, there was a discussion about the benefits and challenges of vaccination and these challenges include barriers to vaccine delivery and vaccine uptake. Concerning antimicrobial resistance, individuals tend to use these agents to cure diseases. This has the potential to cause resistance and various strategies are thus necessary in order to overcome antimicrobial resistance.

## Workshop report

### "We are not able to predict major disease outbreaks"

The recent disease epidemics (Ebola, Nipah virus, Lassa virus and continued flu epidemic) are a clear indication that we cannot predict major disease outbreaks. The Ebola Virus Disease (EVD) was first discovered in 1976 near the Ebola River in the DRC. EVD is classified as a zoonotic disease since it emanates from animals and then passed on to people. This disease is common in the DRC with the current outbreak being the ninth one. The Ebola epidemic was recently detected on the 8^th^ of May 2018 and up to the 6^th^ of July, there has been a total of 53 Ebola cases with 38 confirmed and 15 probable cases, that is, the suspected cases that died without having been confirmed in the laboratory [5]. The Nipah virus outbreak in India is another example that diseases can emerge without having been predicted. This virus has so far claimed 17 lives, with one person having survived the disease. There is no known vaccine or therapy to treat or prevent Nipah, therefore, healthcare workers use supportive treatment while the immune system tries to fight against this virus [2].

Another disease outbreak, that is, Lassa fever, was detected in 19 states of Nigeria in January 2018 where it claimed 107 lives from 423 confirmed cases. In addition, 27 healthcare workers have been infected and eight have died. As for influenza, its detection continues to rise in the Southern part of the African continent and in other countries such as Australia and New Zealand. The WHO laboratories have isolated more than 52621 suspected cases of flu, of which 1376 were confirmed positive for influenza virus. Of these positive cases, 1047 were type A and 329 type B influenza. Since the disease outbreaks cannot be detected, response plans need to be developed and implemented when these outbreaks occur [6]. We are not able to detect major disease outbreaks, however, we can prepare in advance to counter the emergence of diseases. Preparing for the emergence of diseases include developing preparedness or response plans, by, for example, creating a repository of microbial agents, conducting a functional analysis of virus families causing diseases like flu and implementing the pandemic plans [6]. Furthermore, bridging the gap between zoonotic experts and policy makers is one of the very important strategies to consider if we are to win the fight against a certain disease epidemic. We should also not forget the benefits vaccination has brought to humankind as far as fighting infections is concerned [7].

As per the discussions in the OHC, veterinary vaccines are also very important to develop in order to prevent disease transmission from animals to humans. Vaccination, has, in the past, prevented and fought diseases like smallpox, polio, measles, and whooping cough. Therefore, it is important for experts to always emphasize the importance of vaccination and the consequences that come with choosing not to vaccinate [7]. This implies that the One Health Community should be committed to enthusiastically communicate about the need for individuals to vaccinate.

### Challenges around vaccination

Although vaccination is a worldwide tool used to prevent diseases, there are still challenges around it. These include barriers to vaccine delivery, e.g. cold chain issues and lack of trained staff and poor healthcare infrastructure. Cold chain issues may involve, storage activities (interrupted refrigeration), cold packaging and distribution factors. On the other hand, lack of trained staff and poor healthcare infrastructure are major barriers that could decrease vaccination coverage tremendously if attention is not paid towards fixing such problems [8].

Other barriers include those pertaining to vaccine uptake, e.g. affordability, trust of healthcare providers and sociocultural factors. Although the cost of vaccines per child has increased, the cost-effectiveness of immunization in low and middle-income countries has made vaccines easily accessible and hence has contributed towards increased coverage rates. Another important factor that determines vaccine uptake is the manner in which healthcare providers present themselves and communicate about vaccines. There tends to be some resistance towards taking vaccines if the recipient does not trust or believe in the provider. Regarding sociocultural issues, the lack of understanding on what has caused the disease and also religious and social restrictions enforced by religious and community leaders on vaccination have a negative impact on vaccination [9].

### Antimicrobial resistance

Antimicrobial resistance (AMR) is the capability of microorganisms, e.g. bacteria, viruses and some parasites, to stop antimicrobials (e.g. antibiotics, antivirals and antimalarials) from working against that particular pathogen [10]. There generally has been an emphasis or focus on promoting antimicrobials, e.g., by making them less costly. This implies that people will tend to use these agents to cure diseases. This can lead to the overuse of antimicrobials which can cause resistance; therefore, various strategies are necessary in order to overcome this problem. These strategies include pushing for the use of vaccines by demonstrating the cost benefits of both animal and human vaccines and addressing high costs in low and middle-income countries [11]. These strategies would encourage or lead to the use of vaccines to prevent disease occurrences and possibly also decrease the use of antimicrobials.

## Conclusion

The recent emergence of the diseases in the various countries clearly shows that we cannot predict outbreaks, however, developing preparedness or response plans could play a significant role in managing and controlling the spread of disease. Developing response plans does not only involve strategies such as creating a repository of microbial agents, conducting a functional analysis of virus families causing diseases but should also include implementing the pandemic plans in order to successfully fight against the disease outbreaks. In addition to developing response plans, there should be a bridge between zoonotic experts and policy makers to successfully fight against emerging diseases. We should also support the use of vaccination because of the benefits it has brought to humankind as far as fighting infections is concerned. Although vaccination is a significant public health tool used to prevent diseases, it has several challenges which include vaccine delivery (e.g. cold chain issues) and vaccine uptake (e.g. affordability, trust of healthcare providers and sociocultural issues). It is very important to work towards overcoming these vaccination challenges as promoting the use of vaccines has the potential to help fight against antimicrobial resistance. The overuse of antimicrobials can lead to resistance; therefore, the One Health Community can show that vaccination has cost benefits; this is one of the greatest strategies to fight against antimicrobial resistance.

## Competing interests

The author declares no competing interests.
